# Canadian residents’ perceptions of cross-cultural care training in graduate medical school

**Published:** 2017-12-15

**Authors:** Barinder Singh, Emma Banwell, Dianne Groll

**Affiliations:** 1 Queen’s University, Ontario, Canada; 2 Department of Psychology, Queen’s University, Ontario, Canada; 3 Department of Psychiatry, Queen’s University, Ontario, Canada

## Abstract

**Background:**

The Royal College of Physicians and Surgeons of Canada specifies both respect for diversity as a requirement of professionalism and culturally sensitive provision of medical care. The purpose of the present study was to evaluate the perception of preparedness and attitudes of medical residents to deliver cross-cultural care.

**Methods:**

The Cross Cultural Care Survey was sent via e-mail to all Faculty of Medicine residents (approx. 450) in an academic health sciences centre. Comparisons were made between psychiatry residents, family medicine residents, and other residency groups with respect to training, preparedness, and skillfulness in delivering cross-cultural care.

**Results:**

Seventy-three (16%) residents responded to the survey. Residents in psychiatry and family medicine reported significantly more training and formal evaluation regarding cross-cultural care than residents in other programs. However, there were no significant differences in self-reported preparedness and skillfulness. Residents in family medicine were more likely to report needing more practical experience working with diverse groups. Psychiatry residents were less likely to report inadequate cross-cultural training.

**Conclusion:**

While most residents reported feeling skillful and prepared to work with culturally diverse groups, they report receiving little additional instruction or formal evaluation on this topic, particularly in programs other than psychiatry and family medicine.

## Introduction

There is a growing body of literature examining the impact of sociocultural factors such race and ethnicity on health and clinical care, and the need to address cultural safety in healthcare.^1^–^4^

Training in cultural safety has risen to the forefront of medical education in part because Canada is becoming increasingly culturally diverse. According to a 2015 Statistics Canada report, approximately 20% of the Canadian population currently belongs to a visible minority and this will increase to one in three by 2031, with one in four Canadians being foreign-born.^5^ In addition to being able to provide culturally sensitive medical care to all immigrants to Canada, the Canadian First Nations, Inuit, and Metis populations also have their own cultures, beliefs, and values regarding medical care that need to be taken into consideration.

The importance of formally training physicians to deliver care to diverse patient populations has been widely advocated.^3^,^6^–^9^ Sociocultural differences between patients and physicians can influence communication and clinical decision making,^10^,^11^ and there is evidence that patient-physician communication is directly liked to patient satisfaction, adherence, and overall quality of care.^12^,^13^ When sociocultural differences between patients and physicians are not understood or addressed, patients can be left dissatisfied,^11^,^14^,^15^ less likely to adhere to treatments, which can result in poorer health outcomes.^16^,^17^.

The need for health care professionals to effectively provide care to culturally diverse populations has resulted in the development of formal educational initiatives in “cross cultural care.”^17^ For example, CanMEDs^18^, the physician competency framework put forth by the Royal College of Physicians and Surgeons of Canada, specifies respect for diversity as a requirement of professionalism, and states under the role of Communicator that physicians should “facilitate discussions with patients and their families in a way that is respectful, non-judgmental, and culturally safe.”^18^ A 2010 review of 14 English Canadian medical undergraduate schools revealed that while all schools did provide cultural diversity education, each school developed their own course content and pedagogical approach resulting in several different executions and definitions of cultural diversity education.^19^ However the overall concept of cultural diversity education can be generally described as education that focuses on improving clinicians’ abilities to understand, communicate with, and provide quality care to patients from diverse backgrounds.^20^

Given the requirement for cultural diversity training in North American medical schools, there is a paucity of information on the effectiveness of this training or the perception of medical trainees on their preparedness to provide culturally diverse care. One 2005 study of 2047 United States (US) medical residents in their final year of study revealed that they reported feeling much less prepared to provide culturally sensitive care than other technical and clinical care.^20^,^21^ A second study done in 2010, comparing surgery residents’ and family medicine residents’ perception of cross-cultural training found that while family medicine residents received more training and felt more prepared to deliver cross-cultural care, surgery residents believed they would be more skillful.^22^ A second US study found links between knowledge and increased feelings of skillfulness and decreased feelings of helplessness. However, as resident knowledge increased through training, the more helpless they reported feeling in their ability to deliver cross-cultural care.^23^ The authors suggest that “while further study of the nature of this relationship is needed, one potential implication is that different types of training may be needed to increase residents’ perceived knowledge. Alternatively, the finding could indicate that more training increases residents’ awareness of the limits of their knowledge and the possibilities of encountering challenges in providing cross-cultural care.”^23^

Only one group of researchers has published on cultural sensitivity training in Canadian medical schools.^24^,^25^ In 2000 Azad et al.^24^ used structured telephone interviews to obtain information regarding cultural sensitivity training from 16 Canadian medical schools. School officials were interviewed regarding the schools’ objectives, curriculum, methods and evaluation regarding cultural sensitivity training. The authors concluded that “…while progress has been made, lack of adequate resources and a number of obstacles to inclusion of multicultural health content in curricula appear to remain ongoing problems.”^24^

A second report by Azed et al. published in a newsletter in 2004,^25^ describes a survey of Canadian medical residents and medical students regarding cultural sensitivity training. Unfortunately, beyond describing the participants as medical students and residents, there is no information regarding the survey population, the questions asked, or the survey methodology. However, the authors reported that while both medical residents and medical undergraduate students believed cultural sensitivity training was very important, they did not feel well enough prepared to deal with patients of culturally diverse backgrounds.^25^

Given the paucity of information regarding cultural-sensitivity training and the readiness of Canadian residents to provide cross-cultural care, the objective of this study was to assess the self-perceived preparedness of resident physicians at a Canadian academic institution to provide cross-cultural care. This Canadian study compares, using a validated measure of cross-cultural training^20^,^23^ the perceived readiness, comfort, and skill level of psychiatry, family medicine, and other residents to provide cross-cultural care.

## Methods

The present study is a cross-sectional online survey. All medical residents in 11 specialties in all years of residency training in the Queen’s University Faculty of Health Sciences were eligible for the study. The residents were sent an e-mail asking them to participate in the study with a link to an online version of the Cross Cultural Care Survey (CCCS)^20^ hosted by Fluid Surveys.

The CCCS consisted of 45 questions including basic demographic information and a slightly modified (for Canadian use) version of the CCCS (see Appendix A). The CCCS was developed by Weissman et al.^20^ to assess resident preparedness to treat culturally diverse patient populations. The CCCS assesses preparedness, skill, attitudes, and amount of cultural instruction in resident training programs. Specifically, in addition to demographic information, the CCCS covers four areas of cross-cultural care delivery preparedness: identification of any additional training received in residency, perceived preparedness to deliver cross-cultural care, availability of resources (such as translators) to deliver cross-cultural care, and perceived personal skill level in delivering cross-cultural care. The CCCS has demonstrated reliability and validity and is easy to complete.^23^

Demographic information collected included gender, self-identified race or ethnic background, if born in Canada, attended a Canadian medical school, or had received medical training outside of Canada, if spoke languages other than English, and year of residency.

### Data analysis

Survey data were collected online and downloaded into SPSS v.22 for analysis. Demographic and descriptive analysis were conducted using frequency counts and percentages, Mann-Whitney U tests were conducted to compare the response to each question by respondents’ gender, if they graduated from a Canadian medical school, if they worked or had additional training outside of Canada, and if they were born in Canada. Kruskall-Wallis tests were used to compare the responses of three residency groups. Multiple comparisons were controlled for using a Bonferroni correction, and missing data were treated as missing and statistical significance was set at p<0.05

### Ethics

Prior to commencement, this study was approved by Queen’s University Health Sciences and Affiliated Teaching Hospitals Research Ethics Board.

## Results

Approximately 450 medical residents (an exact number was not available due to residents who were on leave, or had switched schools, or were unavailable for other reasons) were sent a link to the survey via their medical school e-mail addresses, and a total of 73 residents responded (an approximate response rate of 16%). Fifteen respondents were in the Psychiatry residency program (20.5%), 24 in Family Medicine (32.9%), and the remaining 34 residents (46.6%) were in “other” residency programs (anesthesia (2), emergency medicine (3), ophthalmology (2), pathology (2), pediatrics (1), public health (2), radiology (1), not specified (10)).

Table 1 shows the demographic characteristics by residency program. Residents were then grouped into “psychiatry,” “family medicine,” and “other” and responses to the CCCS questions were compared across the three groups.

Table 2 shows the mean scores from the CCCS questions regarding the amount of ***additional*** ***training*** they received in cross-cultural care: “Besides what you learned in medical school, how much additional instruction during your residency has been devoted to teaching you the cross-cultural aspects of various cross-cultural skills?” Responses ranged from 0 = no additional training to 3 = a lot of additional training. There were no statistically significant differences in additional training between psychiatry residents and family medicine residents, however residents in psychiatry and family medicine had statistically significantly more instruction in all areas of cross-cultural skill than residents in other programs. Overall, the majority of responses had a mean score of less than 2/3 indicating only “very little” to “some” additional training during residency.

Table 3 shows how residents rated how ***skillful*** they felt they were to be able to provide cross-cultural care with respect to ten different aspects of care such as taking a history, identifying customs or beliefs that may affect care, comprehension of English, and understanding the cause of their illness. Answers ranged from 1 (not at all) to 5 (very skillful). There were no significant differences between residency programs in perceived skillfulness with average scores ranging from 2.86 to 4.04, moderate to very skillful.

Similarly, Question 4 (Table 4) asked residents to answer “How ***prepared*** do you feel to care for patients in a variety of situations” with responses ranging from 0 (very unprepared) to 4 (very well prepared). Again, there were no significant differences between residency programs in perceived preparedness. However, mean scores in preparedness ranged from 1.63 to 2.47, somewhat unprepared to somewhat prepared.

Table 5 shows the results from the question “How much of a problem is each of the following when delivering cross-cultural care” followed by eight resources or barriers, and scored on a scale of 0= no problem to 3= big problem. Table 5 shows that “Lack of practical experience in caring for diverse populations” was a significant problem for family medicine residents compared to psychiatry and other programs. Similarly, “inadequate cross-cultural training during residency” was a significantly greater problem for family medicine and other programs than for psychiatry residents. The biggest problems identified by the residents across all programs were “Poor access to written materials in other languages” and “poor access to medical interpreters.”

Residents were also asked how often they had been formally assessed with respect to their ability to handle cross-cultural issues and doctor-patient communication, (0=none, 3=a lot) there was no statistically significant difference between the groups (p=0.068) however, psychiatry and family medicine residents were evaluated slightly more often than those in other programs (mean, SD, respectively) (1.13 (1.06), 1.17, (0.96), 0.64 (0.90)). Overall the results show that there is very little formal evaluation taking place in any program.

Residents were asked to rate the following six experiences during residency for their usefulness in treating culturally diverse populations: lectures and seminars, case-based discussions, on-the-job-training in community based settings, on-the-job training in hospital settings, the diversity of colleagues, and having faculty as role models. There were no statistically significant differences in how useful the three resident groups found the six identified experiences, with the exception of case based discussions. Psychiatry residents were more likely to find case-based discussions “very useful” than other residents (p=0.021). It is interesting to note that 35% and 29% of “other” residents reported receiving no lectures or seminars or case-based discussions respectively, compared to 7% of psychiatry and 8% of family medicine residents.

Finally, residents were asked “How important do you feel it is for physicians in your specialty to consider the patient’s culture when providing care?” and “How important is it to you to practice in a setting that has a diverse racial and ethnic patient mix?” Ninety-three percent of residents responded that it is moderately to very important to consider patients culture when providing care and 70% of residents thought it was important to practice in a setting that has a diverse racial and ethnic patient mix.

Finally, we re-analyzed the responses to the questions and compared responses by country of birth (Canada or not), location of training (Canada or not) and provision of medical care outside of Canada (yes/no). Individuals born outside of Canada had, on average, significantly more training on the items identified in Table 2 (p=0.006) than those born in Canada mean (SD) score 1.81/3 (0.70) vs 1.33/3 (0.63). There were no overall statistically significant differences between individuals who had medical training outside of Canada or had provided medical care outside of Canada, nor any overall differences between graduates of a medical school within Canada or a school outside of Canada.

## Discussion

This study builds upon and contributes to the work of Weissman et al.,^20^ Chun et al.,^22^,^23^ and Azad et. al.^25^ in their studies of cross-cultural training in residency. This study shows that while psychiatry and family medicine residents report receiving additional formal instruction in the aspects of cross-cultural care, other residency programs do not report receiving additional formal instruction. Interestingly, residents who were born outside of Canada, regardless of their program, report significantly more cross-cultural training in their residency than those born in Canada. This finding is tempered by the fact that neither group reports more than ‘very little’ additional instruction on average.

Results also show that residents are not receiving formal assessment of their cross-cultural skills, however, despite these findings, residents in all programs in this study reported feeling equally and sufficiently skilled in their ability to care for patients as well as equally and sufficiently prepared. Chun et al.^22^,^23^ also report that despite residents’ reporting little additional formal cross cultural training in residency, and rating themselves as being, on average, only ‘somewhat prepared’ to provide cross-cultural care, they still rated themselves highly on their skillfulness to deliver cross-cultural care.

Possible explanations for this finding could be that residents feel they have acquired sufficient cultural skills without formal training. This could be reflected by the cultural and ethnic diversity of our study sample, with approximately 56% being able to speak a language other than English, and almost half (48%) having had medical training or provided medical care outside of Canada. However, when responses were examined with respect to residents’ location of medical school training, additional training or medical practice, or even if they were born in Canada, there were very few differences in responses between individuals who had graduated from a Canadian medical school or had previous training or experience outside of Canada. In fact, residents born outside of Canada reported significantly more cross cultural training – this may be a “reversal” of what we think when studying cross cultural training in that residents born outside of Canada may receive what they see as additional training to work with Canadians.

Overall the greatest issues are lack of resources, specifically poor access to written materials in other languages and poor access to medical interpreters. Although no singular problem scored above a “moderate problem” rating, there were some discrepancies between problems in different programs. Residents in family medicine found lack of practical experience in caring for diverse patient populations and inadequate cross-cultural training during residency to be a greater problem than psychiatry or other programs. This reveals that although these are not severe problems, more instruction may need to be directed at family medicine residents in terms of practical experience and training in cross cultural care.

There are several limitations to this study include a small sample size, very low response rate, and being restricted to one academic health sciences centre. As such, the results from this study need to be interpreted with caution, and the generalizability of these results may be limited. Future research should attempt to replicate these findings with larger groups, better response rates, as well as in different geographical areas. Demographics from this study reveal that residents were an ethnically diverse group with many individuals being born, educated, or providing care, outside of Canada. These demographic factors may have had an effect of residents’ perceived skillfulness and preparedness in cross cultural care. Finally, residents completed self-report measures of skillfulness and preparedness which may not be reliable. Future research may also include other measures of skill and preparedness such as grades or reports from supervisors or patients.

However, as this is presently the only study of Canadian residents’ cultural-sensitivity training and the readiness of Canadian residents to provide cross-cultural care there are clearly many opportunities for future research. Ideally, Canadian medical schools would institute routine annual evaluations of their cross-cultural training, and this information could be shared and published at regular intervals. This study is of one site only, so future research studies need to examine several sites. This study had very limited responses from residents outside of psychiatry and family medicine, future research needs to take a closer look at other residency specializations.

### Conclusion

Findings from this study reveal that residents feel skilled and prepared in terms of caring for their patients, despite not having a great deal of additional instruction or evaluation of cross cultural care in residency. Future programs should institute uniform cross cultural instruction and formal and robust assessment in all residency programs in order to determine if perceived skillfulness and preparedness translates into actual practice. Programs may also include making cross cultural resources, materials, and interpreters more readily available for residents in all programs. The results from this study provide an important baseline to which future research can be compared. With the increasing cultural diversity in Canada the need for Canadian physicians to be well trained in culturally sensitive delivery of healthcare with appropriate resources in place cannot be understated.

## Figures and Tables

**Table 1:** Demographics

Gender	Psychiatry N (%) n=15	Family Medicine N (%) n=24	Others N (%) n=34

Male	6 (40)	4 (19)	7 (27)
Female	9 (60)	17 (81)	19 (73)

**Race/Ethnicity**			

White	3 (20)	13 (62)	15 (58)
Black	1 (7)	0	1 (4)
Asian	6 (40)	2 (10)	6 (23)
East Indian	2 (13)	5 (24)	2 (8)
Hispanic	0	1 (5)	0
Other	3 (20)	0	2 (8)

**Born in Canada**			

Yes	5 (33)	13 (62)	22 (85)
No	10 (67)	8 (38)	4 (15)

**Graduate of a Canadian Medical School**			

Yes	5 (33)	13 (62)	24 (92)
No	10 (67)	8 (38)	2 (8)

**Speak any languages other than English?**			

Yes	13 (86.7)	12 (60)	16 (61.5)
No	2 (13.3)	8 (20)	10 (38.5)

**Medical Training or provided care outside of Canada?**			

Yes	13 (87)	12 (57)	10 (38)
No	2 (13)	9 (43)	16 (62)

**Year of Residency**			

Year 1	2 (13.3)	8 (38.1)	5 (19.2)
Year 2	3 (20)	10 (47.6)	6 (23.1)
Year 3	3 (20)	3 (14.3)	6 (23.1)
Year 4	6 (40)	0	6 (23.1)
Year 5	1 (6.7)	0	3 (11.5)

**Table 2:** Besides what you learned in medical school, how much additional instruction during your residency has been devoted to teaching you the cross-cultural aspects of...your residency has been devoted to teaching you the cross-cultural aspects of...

Cross-Cultural Skill	Psychiatry Mean (SD)	Family Medicine Mean (SD)	Other Mean (SD)	*p*
Determining how a patient wants to be addressed and interacted with?	1.93 (1.03)	1.58 (0.83)	0.94 (0.85)	0.001
Taking a social history	2.47 (0.92)	2.21 (0.83)	1.47 (0.86)	<0.001
Assessing a patient’s understanding of the causes of his or her illness	2.20 (1.01)	2.08 (0.83)	1.53 (0.83)	0.012
Negotiating a treatment plan	2.43 (0.65)	2.25 (0.80)	1.56 (0.99)	0.003
Identifying mistrust of the system or physician	1.57 (0.94)	1.38 (0.82)	0.88 (0.73)	0.018
Identifying ability to read or write English	1.79 (0.98)	1.21 (0.88)	0.68 (0.54)	<0.001
Identifying religious beliefs that might affect care	1.93 (0.92)	1.38 (0.65)	1.12 (0.73)	0.008
Identifying cultural customs that might affect care	1.79 (1.12)	1.42 (0.72)	0.94 (0.69)	0.010
Identifying how patient makes decisions with family	2.00 (0.96)	1.58 (0.78)	1.06 (0.92)	0.005
Delivering services through a medical interpreter	1.50 (1.09)	1.25 (0.79)	0.82 (0.80)	0.049

Scale: 0=None, 1=Very Little, 2=Some, 3=A Lot.

**Table 3:** How skillful do you feel to care for patients (or pediatric patients’ families)…

Item	Psychiatry Mean (SD)	Family Medicine Mean (SD)	Other Mean (SD)	*p*
Determining how a new patient wants to be addressed	3.73 (1.03)	3.57 (0.60)	3.48 (1.01)	0.688
Taking a social history	3.79 (1.12)	4.00 (0.71)	4.04 (0.74)	0.642
Assessing a patient’s understanding of the causes of illness	3.47 (0.92)	3.86 (0.48)	3.76 (0.93)	0.340
Identifying mistrust of the system or physician	3.27 (1.03)	2.90 (0.63)	3.04 (0.91)	0.461
Negotiating a treatment plan	3.60 (0.99)	3.81 (0.40)	3.80 (0.71)	0.624
Identifying ability to read & write English	3.00 (0.85)	2.86 (0.79)	2.96 (0.89)	0.866
Identifying religious beliefs that might affect care	3.21 (0.98)	2.90 (0.77)	2.92 (0.81)	0.503
Identifying cultural customs that might affect care	3.07 (1.16)	3.10 (0.94)	3.20 (0.91)	0.715
Identifying how patient makes decisions with family	3.00 (0.96)	3.19 (0.87)	3.12 (1.09)	0.899
Delivering services through a medical interpreter	3.73 (1.03)	3.57 (0.60)	3.48 (1.01)	0.856

1=Not at all Skillful, 5=Very Skillful.

**Table 4:** How prepared do you feel to care for patients (or paediatric patients’ families…)

Item	Psychiatry Mean (SD)	Family Medicine Mean (SD)	Other Mean (SD)	*p*
From cultures different from own	2.47 (0.99)	2.21 (0.72)	2.45 (0.83)	0.508
With health beliefs at odds with Western medicine	2.27 (1.10)	2.00 (0.66)	1.93 (0.80)	0.440
With a distrust of the Canadian health system	2.13 (1.30)	1.63 (0.71)	1.69 (0.93)	0.206
With limited English proficiency?	2.27 (1.63)	1.96 (0.75)	1.93 (0.92)	0.492
Who are new immigrants	2.27 (1.03)	1.79 (0.78)	2.03 (0.82)	0.241
Whose religious beliefs affect treatment	2.00 (1.07)	1.58 (0.72)	1.83 (0.89)	0.333
Who use alternative/complementary medicine	2.13 (1.25)	2.00 (0.83)	1.86 (0.96)	0.670
Who are members of racial and ethnic minorities	2.40 (1.21)	2.46 (0.78)	2.45 (1.09)	0.983

Scale: 0=Very Unprepared, 1=Somewhat Unprepared, 2=Somewhat Prepared, 3=Well Prepared, 4=Very Well Prepared.

**Table 5:** How much of a problem is each of the following when you are delivering cross-cultural care?

Item	Psychiatry Mean (SD)	Family Medicine Mean (SD)	Other Mean (SD)	*P*
Lack of practical experience in caring for diverse patient populations	1.00 (0.93)	1.57 (0.51)	1.19 (070)	0.029
Lack of time to adequately address cultural issues	1.20 (0.78)	1.70 (0.70)	1.77 (0.91)	0.085
Inadequate cross-cultural training during residency	0.73 (0.80)	1.39 (0.78)	1.23 (0.77)	0.024
Poor access to medical interpreters when they are needed	1.60 (1.18)	2.22 (0.95)	2.19 (0.94)	0.134
Poor access to written materials in other languages, including health education pamphlets, consent forms, etc.	1.86 (0.95)	2.26 (0.86)	2.00 (1.06)	0.431
Absence of good role models or mentors for cross-cultural care among the faculty	0.73 (0.70)	1.22 (0.80)	1.04 (0.72)	0.155
Dismissive attitudes about cross-cultural care among attending physicians	0.93 (0.88)	0.87 (1.01)	0.77 (0.77)	0.838
Dismissive attitudes about cross-cultural care among your fellow residents	0.87 (0.92)	0.74 (0.97)	0.65 (0.69)	0.743

Scale: 0=No problem, 1=Small problem, 2=Moderate problem, 3=Big problem
